# Research on visual search strategies during apparatus throw-and-catch in group rhythmic gymnastics

**DOI:** 10.3389/fpsyg.2025.1700617

**Published:** 2025-11-05

**Authors:** Chuncen Zhou, Yujun Cai, Kai Li, Xinmiao Zhang, Changhao Tang

**Affiliations:** ^1^School of Physical Education, Shanghai University of Sport, Shanghai, China; ^2^College of Sports Industry and Leisure, Nanjing Sport Institute, Nanjing, Jiangsu, China

**Keywords:** rhythmic gymnastics, visual search, eye-tracking, apparatus exchange, task difficulty

## Abstract

**Objective:**

This study aimed to investigate the visual search characteristics and strategies of expert rhythmic gymnasts during apparatus throw-and-catch tasks under different apparatus types and difficulty levels, in order to provide theoretical support for optimizing routine choreography and designing effective visual training programs.

**Methods:**

Fifteen rhythmic gymnasts at or above the national first-class level were recruited. Tobii Pro Glasses 3 wearable eye tracker was used to collect eye movement data during four throw-and-catch tasks of varying difficulty levels (Hoop 1–3, Ball 4). Global eye movement metrics (total duration of whole fixations, number of whole fixations, number of saccades) and area of interest (AOI) indicators (total duration of fixation, total duration of visit, total duration of glances, etc.) were analyzed using Kruskal–Wallis tests and Bonferroni-corrected post-hoc comparisons.

**Results:**

As task difficulty increased, athletes showed significantly longer total fixation duration, more fixation points, and more saccades (*p* < 0.001, *η*^2^ = 0.105); Under the same difficulty level, hoop-related tasks imposed a higher visual search load compared to ball tasks (*p* < 0.05); AOI analysis indicated significantly greater visual attention during the catching phase than the throwing phase. In high-difficulty conditions, athletes demonstrated more efficient visual strategies, such as shorter fixation times and reduced saccade paths.

**Conclusion:**

Expert rhythmic gymnasts actively adjust their visual strategies based on task complexity and apparatus characteristics, showing goal-oriented behaviors and evidence of experience transfer. Visual search efficiency is jointly influenced by movement difficulty and apparatus type. It is recommended that future training incorporates task-specific visual training programs to enhance throw-and-catch success rates and overall performance.

## 1 Introduction

Rhythmic gymnastics is a complex sport characterized by open-loop control (Newell, 1991), in which both the difficulty of routine composition and the quality of execution directly affect athletes’ competitive performance. The discipline includes both individual and group events. In group routines, the difficulty score is composed of four components: body difficulty with or without exchange (DB), exchange difficulty (DE), dynamic elements with rotation (R), and collaboration difficulty (DC). As a hallmark of group routines, exchange elements require athletes to initiate movement simultaneously and throw the apparatus either to a height of twice their body height or over a distance of eight meters to a teammate (Gymnastique FIG, 2025). The base score for an exchange is 0.2. To enhance routine competitiveness, coaches often increase the difficulty of the DE element by incorporating bonus features during the throw, the flight of the apparatus, or the catch, such as rotations, non-hand assistance, floor positions, or out-of-sight receptions. Accurate throwing and catching are crucial for the successful execution of exchange elements; thus, the precision of these movements largely determines their success rate.

In rhythmic gymnastics competitions, athletes must precisely coordinate body difficulties with apparatus techniques (Agopyan, 2014). Typically, the direction of gaze aligns with the focus of visual attention, and compared to head or body orientation, eye position is a more reliable predictor of the object of interest and forthcoming actions (Zohary et al., 2022). During exchange elements in group routines, athletes are required to throw the apparatus accurately to a designated teammate and, after completing a series of movements, rapidly locate and track the apparatus. This process demands athletes to possess advanced visual search abilities and the capacity to judge the precise location and spatial depth of the apparatus (Xu et al., 2018). Visual search efficiency refers to the speed and accuracy with which an individual identifies a specific target (Ribeiro et al., 2021). As a core component in routine composition, the precision and stability of apparatus exchanges directly influence an athlete’s final competition score. Visual search efficiency determines how well athletes can acquire and integrate relevant information within a limited timeframe (Borysiuk and Waskiewicz, 2008), thereby affecting their ability to control the exchange accurately. Before competition, gymnasts must quickly adapt to spatial changes between training and competition venues. The degree of adaptation to the competitive environment directly impacts optimal athletic performance, as environmental changes may disrupt proprioception and impair spatial orientation and motor control (Proske and Gandevia, 2012). Visual search plays a dominant role in proprioceptive and spatial awareness functions (Yizhar et al., 2020). Unlike general neurophysiology, our focus here lies on the mechanisms governing the transmission and transformation of visual information during instrument exchange to support rapid action selection–—encompassing retinal sampling, relay transmission via the lateral geniculate nucleus to early visual cortex, and task-related integration along the dorsal (visio-action) pathway. In this paper, visual strategy denotes the task-specific allocation and sequencing of gaze to support perception-action coupling during implement throwing and catching—specifically, where athletes look (AOIs), when they look (throwing vs. catching phases), and how they acquire information temporally and dynamically (gaze duration, access frequency, saccade rate) (Nagano et al., 2004). We operationalize this into overall metrics (TDWF, NWF, NS) and AOI-level metrics (TFD, NOV, TDV, TDG). Effective visual search not only improves the success rate of entire routines but also reduces reaction time during the throw-and-catch process, allowing for more cohesive and compact choreography.

The pattern recognition hypothesis posits that every perceived object is stored as a “template” in long-term memory (Shugen, 2002). When athletes receive environmental information, they match the incoming data with previously stored templates retrieved from memory (Eysenck and Keane, 2020), thereby executing the most optimal movement pattern. As an intermediary variable linking environmental information extraction and motor execution, visual search plays a multidimensional regulatory role in dynamic sports contexts (Vitor de Assis et al., 2020). From the 1950s to the 1960s, Yarbus (2013) pioneered the use of eye movement recordings to study saccadic behaviors when viewing complex images. With technological advancement, modern eye-tracking systems—such as the Tobii Pro series—utilize video and infrared optical technology to capture both pupil and corneal reflections, thereby calculating gaze points and eye movement trajectories (Guo et al., 2024). The continuous evolution of eye-tracking devices has driven deeper research into athletes’ visual search capabilities during sports performance. To date, a substantial body of literature has investigated visual search strategies across various sports, including basketball (Uchida et al., 2014), soccer (Savelsbergh et al., 2005), and table tennis (Piras et al., 2016). These studies have consistently shown that experienced athletes adopt effective visual search strategies to predict the outcomes of moving objects (Ishibashi et al., 2010). For instance, Kim and Lee (2006) examined six elite soccer goalkeepers with over 10 years of experience and found that gaze duration varied between successful and unsuccessful penalty saves, with shorter total fixation durations observed during successful saves. Similarly, Morgan et al. (2009) compared the visual search strategies of elite athletes from different sports and reported significant differences in oculomotor performance and adaptive search behaviors, likely attributable to varying sport-specific demands and cognitive strategies. Ramyarangsi et al. (2024) compared the unique visual processing patterns of elite female athletes. Visual P300 ERP responses were elicited using the visual oddball paradigm, revealing that gymnasts exhibited the shortest visual reaction time (RT) among athletes in gymnastics, football, and esports. Bürger et al. (2022) compared the training effects of VR environments versus the real world for novice balance beam tasks, finding that VR training proved equally effective as real-world training in enhancing the quality of balance beam movements. These findings indicate that visual search strategies differ across sports, influenced by the unique characteristics of each discipline and the athletes’ cognitive processing styles. Moreover, although visual search research in sports such as soccer, basketball, and volleyball provides useful points of reference, its conclusions cannot be directly generalized to group rhythmic gymnastics. Rhythmic gymnastics routines require not only a high degree of spatiotemporal coordination among multiple athletes, but also precise synchronization with musical rhythm and artistic presentation, while complex apparatus manipulations (e.g., throwing, catching, rolling) must be completed within strict time limits (Loquet, 2016). This multilayered integration of esthetic expression, rhythmic control, and precise object manipulation distinguishes rhythmic gymnastics from sports that primarily emphasize tactical decision-making and the execution of actions under dynamic opposition. It is precisely this distinctiveness that highlights the necessity of dedicated research on visual search strategies specific to rhythmic gymnastics, rather than simply applying conclusions drawn from other sports. Understanding the visual search strategies adopted by rhythmic gymnasts during apparatus throw-and-catch in group routines can help athletes optimize their exchange performance and enable coaches to develop more effective and scientifically informed routine compositions.

In summary, this study aims to examine the visual search characteristics of athletes in group rhythmic gymnastics during the apparatus throw–catch process across different apparatus types and difficulty levels. Building on the foregoing theoretical framework and existing empirical work, we hypothesize that as task difficulty increases, the average fixation duration within the throw–catch visual search strategy becomes shorter, and the Number of saccades decreases; furthermore, apparatus type will exhibit between-task differences—the hoop relying more on trajectory monitoring, whereas the ball relies more on spatial localization and rapid oculomotor responses. In addition, we propose that pre-catch dwell, total fixation duration during the throw–catch phase, the Number of whole fixations, and the Number of saccades can serve as practical process indicators for training and monitoring, thereby providing actionable pathways to improve the success rate of complete routines in rhythmic gymnastics.

## 2 Materials and methods

### 2.1 Participants

The G*Power version 3.1.9.7 (Heinrich-Heine-Universität Düsseldorf, Düsseldorf, Germany) was used to determine the appropriate sample size for this study. Power was estimated in G*Power 3.1.9.7 using one-way ANOVA (Cohen’s 𝑓) as a proxy for KW-H (*α* = 0.05, 1–*β* = 0.80, k = 4; 𝑓=0.436; from partial *η*^2^ = 0.16); required 𝑁≈12, achieved 𝑁=15 (Nishiumi et al., 2025). We ultimately included 15 female elite rhythmic gymnasts (3 at the National Master level and 12 at the National First-Class level), with a mean training history of 11.5 years. All athletes had competed multiple times in the National University Rhythmic Gymnastics Championships, achieving runner-up or better placements. Rhythmic Gymnastics is restricted to female competitors in events sanctioned by the International Gymnastics Federation (FIG), hence the research population comprises female athletes.

All participants were right-handed, had normal or corrected-to-normal vision, and reported no color blindness or color weakness. Participation in the study was voluntary, and all subjects provided written informed consent.

Ethical approval was granted by the Shanghai University of Sport Institutional Review Board (Approval No: 102772021RT096).

### 2.2 Instruments

This experiment utilized the Tobii Pro Glasses 3 eye tracker with a sampling frequency of 100 Hz, synchronously recording scene video at 25 frames per second. To ensure the accuracy of the collected data, the experiment was conducted in a well-lit rhythmic gymnastics’ facility with a ceiling height sufficient to meet the requirements of the test. The environment was quiet and free from disturbances, with only the research and testing personnel present to minimize interference from external movement. The apparatus used in the experiment was a rhythmic gymnastics hoop compliant with the standards of the Fédération International de Gymnasium (FIG), ensuring alignment with international competition requirements. The size, weight, and material of the hoop strictly conformed to FIG specifications to ensure the experimental procedures were standardized and the data obtained was reliable.

### 2.3 Indicator selection

Event identification was conducted in Tobii Pro Lab using the Tobii I-VT (Attention) classifier with a velocity threshold of 100°/s: saccades were labeled when eye-in-head velocity stayed above threshold for 20–40 ms, and fixations when eye-in-head angular velocity stayed below threshold for 50–600 ms (Hessels et al., 2018). Fixations were interpreted using head reference. The AOI was dynamic, updated frame-by-frame according to the spatial position of the instrument.

The analysis of eye-tracking indicators in this study was divided into two main parts: first, the entire movement was treated as a single, complete area of interest (AOI); second, based on experimental requirements, each test video was segmented into different AOIs to analyze the participants’ attention allocation during the apparatus throw-and-catch process. Specifically, each video was divided into two AOIs: the throwing phase and the catching phase.

General eye movement indicators: (1) Total duration of whole fixations: the total time an individual’s eyes remain fixated on a specific region during the execution of a visual task. (2) Number of whole fixations: the number of times the eyes pause at different locations while completing the task. Each time the gaze remains focused on a specific area for a minimum duration, it is recorded as a fixation. The identification of a fixation depends on the minimum fixation duration threshold, which is typically set at 80–100 milliseconds. (3) Number of saccades: the number of times the eyes move from one fixation point to another during visual search.

AOI-specific eye movement indicators include the following: Total fixation duration within the AOI, Total duration of Visit, Total duration of Glances, and Number of Visits. These represent, respectively, the time the participant’s gaze remains within a defined AOI, the duration spent moving from one fixation point to another, the total time consumed by saccadic movements, and the total number of visits to the AOI (Holmqvist et al., 2011).

### 2.4 Experimental design and procedure

#### 2.4.1 Experimental design

This study adopted a 2 × 4 (Apparatus × Difficulty) two-factor repeated-measures experimental design. The independent variables comprised two factors: Apparatus (Hoop, Ball); Difficulty: low (0.2 points), medium (0.3 points), high (0.4 points).

Based on the FIG Rhythmic Gymnastics Code of Points for the 2025 cycle, we specified difficulty values for group routines by mapping the selected elements to the table of bonus factors and using their base difficulty values as numeric labels for analysis (Gymnastique FIG, 2025). Accordingly, we constructed three difficulty levels–—low (0.2), medium (0.3), high (0.4)–—each matched one-to-one to the selected elements (e.g., throw–catch, throw–rotation–catch, throw–floor turn–non-hand catch) as prescribed by the Code.

**Figure 1:**
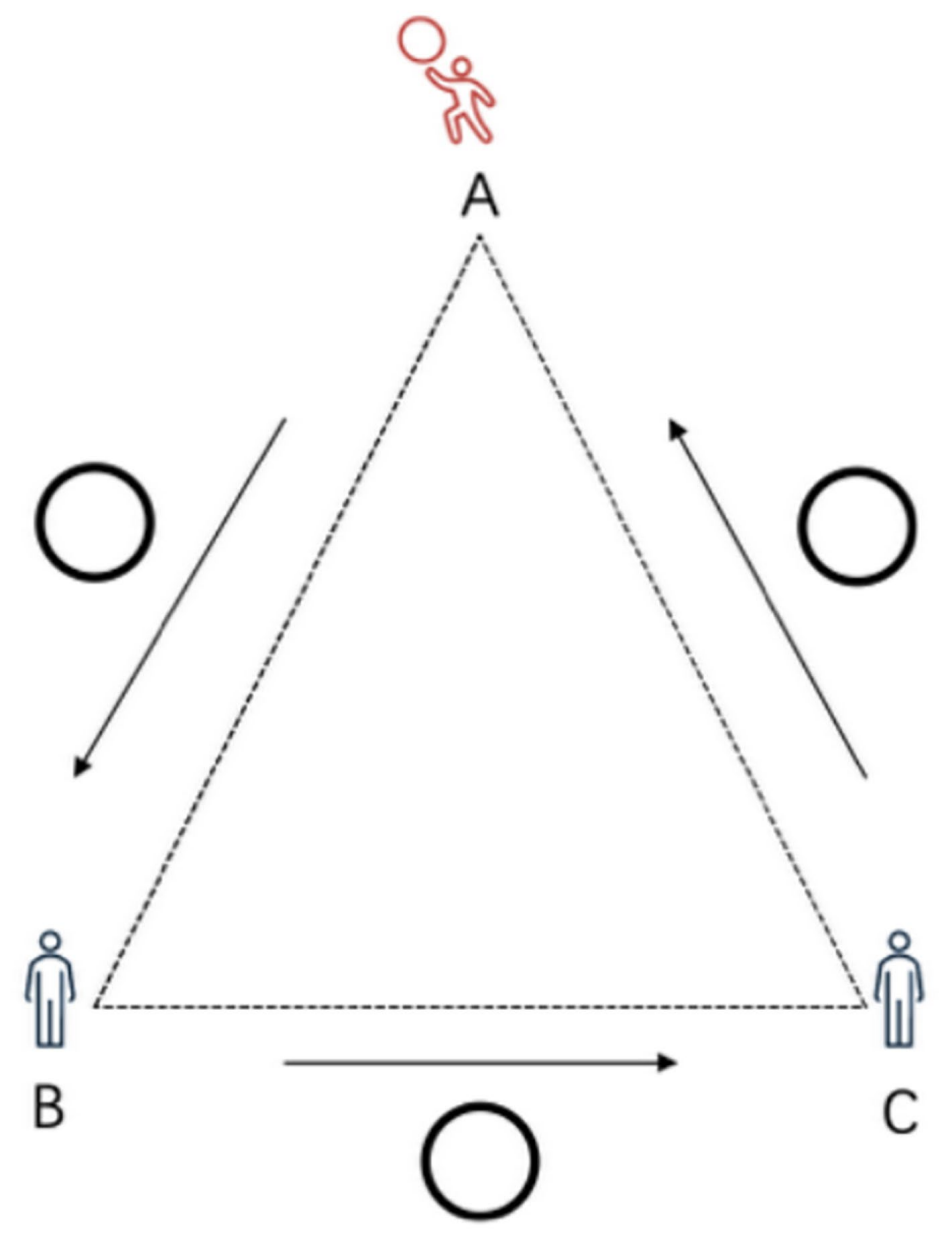
Test position diagram.

The experiment was conducted on a standard 13 m × 13 m rhythmic gymnastics floor. Three points (A, B, and C) were marked at the center of the field, forming an equilateral triangle with 8-meter sides. The participant stood at point A, threw the hoop to the tester at point B to perform the designated test movement, and then received a hoop thrown by the tester at point C. All testers at points A, B, and C began their movements simultaneously upon hearing the start command. According to the experimental design, expert athletes completed four movements as specified in Table 1. To ensure the consistency and accuracy of experimental results, all hoops thrown to participants were delivered by the same athlete, a nationally certified elite gymnast (Figure 1).

**Table 1:** Test movements in the experiment.

Apparatus	Level of difficulty	Movement code	Description	Difficulty score
Hoop	Low	Hoop 1	Throw–Catch	0.2
	Medium	Hoop 2	Throw–Horizontal Rotation–Catch	0.3
	High	Hoop 3	Throw–Floor Rotation–Non-hand Catch	0.4
Ball	High	Ball 4	Throw–Floor Rotation–Non-hand Catch	0.4

Throw–Catch: Starting at position A, the athlete throws the hoop to the teammate at B; subsequently performs a manual (hand) catch of the hoop thrown from C. No body rotation is executed; Throw–Horizontal Rotation–Catch: From A, the athlete throws to B, then performs a stationary upright axial rotation of 360°, and completes a manual (hand) catch of the hoop thrown from C; Throw–Floor Rotation–Non-hand Catch: From A, the athlete throws to B, then executes a 360° turn in a seated position on the floor, and completes a non-manual catch with the lower limb of the hoop thrown from C.

#### 2.4.2 Experimental procedure

(1) Preparation phase: The experiment was conducted at the rhythmic gymnastics gymnasium of Shanghai University of Sport. The venue had sufficient ceiling height and soft lighting. Apart from the research team, no unrelated personnel were present to avoid interference. To ensure the validity and reliability of the results, participants’ basic information was recorded prior to the experiment. The testing procedure, movement requirements, and equipment calibration methods were explained to the participants in detail. They were instructed to perform the test with the same mindset as in official competitions.
(2) Practice phase: After a 10-min warm-up, the testing staff assisted the participants in wearing and adjusting the lenses of the glasses-type eye tracker. An adjustable strap was used to secure the device and minimize measurement errors caused by large movement amplitudes. Once the eye tracker was properly fitted, each participant practiced the designated test movements five times while holding the apparatus, allowing them to adapt to the device before calibration began.
(3) Device calibration: Participants, while wearing the eye-tracking glasses, stood at the designated starting position and focused on a calibration card. When the calibration confirmation tone was heard, they were ready to begin the test. The Tobii Pro Glasses 3 were worn with the factory adjustable head strap; no external fixation system was used.
(4) Formal experiment: The formal testing procedure was the same as in the practice phase. It included four test movements: throw–catch with hoop, throw–rotation–catch with hoop, throw–floor rotation–non-hand catches with hoop, and throw–floor rotation–non-hand catches with ball. Each movement was performed five times. The entire experiment lasted approximately 30 min.

### 2.5 Data processing and statistical analysis

Scene video and synchronized gaze streams recorded with Tobii Pro Glasses 3 were processed in Tobii Pro Lab. After delineating AOIs and running event identification, the software exported the required metrics and raw time series (with timestamps and validity flags), including global metrics TDWF, NWF, NS and AOI-level metrics TFD, NOV, TDV, TDG.

AOI delineation. Throw phase: from the first frame in which the hoop leaves the hand to the first frame in which the trunk initiates axial rotation. Catch phase: from the first frame in which the hoop first appears in the scene-camera image to the first frame of limb–hoop contact. AOIs were dynamic: the apparatus AOI was updated frame by frame according to the hoop’s instantaneous spatial position. To reduce confounding from trial duration on absolute fixation time, we report—besides the Total duration of whole fixations (TDWF)—a fixation-time ratio (FTR), computed as TDWF divided by the duration of the throw-and-catch episode (from apparatus release to first contact).

Given that, for some conditions (ball), the number of difficulty levels was imbalanced and the data distributions violated the assumptions of normality and homoscedasticity, we employed nonparametric methods within the two-factor repeated-measures framework. For both global eye-movement metrics and AOI-based metrics, comparisons were conducted under the Apparatus type × Difficulty level structure; omnibus comparisons across conditions used the Kruskal–Wallis test, with Bonferroni-corrected pairwise *post hoc* tests when significant; *η*^2^ was reported as the effect-size index, and Cohen’s d was computed for post hoc contrasts to assess effect magnitude; Pearson correlation coefficients were calculated to examine the relationships among task difficulty, apparatus type, and eye-movement metrics.

## 3 Results

### 3.1 Participant characteristics analysis

Table 2 summarizes the basic characteristics of the 15 rhythmic gymnasts who participated in the study. In terms of age distribution, the highest proportions were observed at ages 19 and 21, accounting for a combined total of 53.3%. Regarding training experience, 60% of participants had over 11 years of training, indicating a strong athletic background. Height was primarily concentrated between 161 and 170 cm (46.7%), while most athletes weighed between 51 and 58 kg (60.0%). The majority held the National First-Class Athlete certification (80.0%), reflecting a high level of competitive proficiency.

**Table 2:** Participant demographics (*n* = 15).

Variable	Value	Frequency	Percentage
Age (years)	18	2	13.3%
	19	5	33.3%
	20	2	13.3%
	21	3	20.0%
	22	2	13.3%
	23	1	6.7%
Training years	≤5	2	13.3%
	6–10	4	26.7%
	≥11	9	60.0%
Height (cm)	≤160	2	13.3%
	161–170	7	46.7%
	>170	6	40.0%
Weight (kg)	≤50	5	33.3%
	51–58	9	60.0%
	>58	1	6.7%
Athlete level	National First-Class Athlete	12	80.0%
	National Elite Athlete	3	20.0%

### 3.2 Analysis of general eye-tracking indicators

A statistical analysis was conducted on the general eye-tracking data of expert rhythmic gymnasts during apparatus throw-and-catch movements. The results are presented in Tables 3, 4.

**Table 3:** General eye-tracking results during apparatus throw-and-catch under different apparatus/difficulty conditions (M ± SD).

Group	Total duration of whole fixations (ms)			Number of whole fixations (count)			Number of saccades (count)		
	Median	SD	IQR	Median	SD	IQR	Median	SD	IQR
Hoop 1	987	884.68 ± 690.58	1092.25	3.00	4.45 ± 3.49	5.75	2.00	2.23 ± 2.29	3.00
Hoop 2	882	825.77 ± 562.88	901.00	5.00	4.54 ± 2.65	5.00	2.00	2.34 ± 1.51	3.00
Hoop 3	822	903.65 ± 552.80	731.00	5.00	4.79 ± 2.07	3.00	2.00	2.37 ± 1.59	2.00
Ball 4	756	786.46 ± 520.00	528.50	4.00	4.10 ± 2.50	4.00	2.00	2.02 ± 1.50	1.00

Median = 50th percentile; SD, mean ± standard deviation; IQR, interquartile range.

**Table 4:** KW–H and Bonferroni results for overall eye-tracking metrics.

Comparison	Total duration of whole fixations				Number of whole fixations				Number of saccades			
	*z*	*p*	*dᶻ*	*r*	*z*	*p*	*dᶻ*	*r*	*z*	*p*	*dᶻ*	*r*
Hoop 1 vs. Hoop 2	0.104	0.921	+0.03	0.007	−0.331	0.743	−0.09	0.022	−0.923	0.351	−0.24	0.063
Hoop 1 vs. Hoop 3	−3.597	0.000^**^	−0.93	0.244	−4.101	0.000^**^	−1.06	0.278	−3.683	0.000^**^	−0.95	0.250
Hoop 1 vs. Ball 4	0.669	0.506	+0.17	0.045	0.165	0.871	+0.04	0.011	−0.129	0.899	−0.03	0.009
Hoop 2 vs. Hoop 3	−3.634	0.000^**^	−0.94	0.247	−3.576	0.000^**^	−0.92	0.243	−2.899	0.003^*^	−0.75	0.197
Hoop 2 vs. Ball 4	0.615	0.542	+0.16	0.042	0.961	0.335	+0.25	0.065	0.969	0.326	+0.25	0.066
Hoop 3 vs. Ball 4	4.574	0.000^**^	+1.18	0.310	4.941	0.000^**^	+1.28	0.335	4.203	0.000^**^	+1.09	0.285
H	28.842				33.481				25.314			
*p*	<0.001				<0.001				<0.001			
*η*^2^	0.121				0.143				0.105			

*z*, standardized statistic from the Wilcoxon signed-rank test; *p*, Bonferroni-adjusted two-sided *p*-value; dᶻ, within-subject effect size; *r*, rank-based, correlation-type effect size; H, Kruskal–Wallis omnibus test statistic.

#### 3.2.1 Total duration of whole fixations

The Bonferroni *post hoc* pairwise comparison revealed that both apparatus type and difficulty level were significantly associated with the total duration of whole fixations among rhythmic gymnasts (*p* < 0.001, *η*^2^ = 0.121). As shown in Table 4, there was a significant relationship between movement difficulty, apparatus type, and total fixation duration (*p* < 0.001). Further Bonferroni analysis indicated significant differences in total fixation duration between Hoop 1 and Hoop 3, Hoop 2 and Hoop 3, as well as between Hoop 3 and Ball 4. Under the same apparatus condition, increased movement difficulty led to a significant change in total fixation duration (*p* < 0.001), with higher difficulty corresponding to longer total fixation durations. When movement difficulty remained constant but apparatus type differed, expert gymnasts also demonstrated significantly different total fixation durations (*p* < 0.001), with longer durations observed for hoop routines compared to ball routines.

#### 3.2.2 Number of whole fixations

The Bonferroni *post hoc* pairwise comparison showed that both movement difficulty and apparatus type were significantly associated with the number of whole fixations among rhythmic gymnasts (*p* < 0.001, *η*^2^ = 0.143). As shown in Table 4, significant differences in the number of whole fixations were observed across different levels of difficulty and apparatus types, with the Kruskal–Wallis significance test (KW-H) indicating strong statistical significance. Further Bonferroni analysis revealed that, under the same apparatus condition, high-difficulty movements resulted in significantly more fixations than low-difficulty movements (*p* < 0.001). When the difficulty level remained constant but the apparatus type varied, the number of fixations was significantly lower for the ball compared to the hoop (*p* < 0.001).

#### 3.2.3 Number of saccades

The Bonferroni *post hoc* pairwise comparison also indicated that both movement difficulty and apparatus type were significantly associated with the number of saccades (*p* < 0.001, *η*^2^ = 0.105). As shown in Table 4, a significant relationship was found between movement conditions and the number of saccades. Under the condition of differing difficulty scores between Hoop 1 and Hoop 3, an increase in difficulty led to a significant increase in number of saccades (*p* < 0.001). Between Hoop 2 and Hoop 3 (medium vs. high difficulty), expert athletes also exhibited a significant difference in the number of saccades (*p* = 0.003462). In the case of Hoop 3 versus Ball 4—where movement difficulty remained the same but the apparatus differed—the number of saccades for hoop routines was significantly higher than for ball routines (*p* < 0.001).

### 3.3 Analysis of eye-tracking indicators in areas of interest (AOIs)

A statistical analysis was conducted on the eye-tracking data within areas of interest (AOIs) during apparatus throw-and-catch movements by expert rhythmic gymnasts. The results are presented in Tables 5, 6.

**Table 5:** Eye-tracking results in areas of interest under different difficulty/apparatus conditions (M ± SD).

Group	Total fixation duration						Number of visits						Total duration of visit						Total duration of glances					
	Throw			Catch			Throw			Catch			Throw			Catch			Throw			Catch		
	Median	SD	IQR	Median	SD	IQR	Median	SD	IQR	Median	SD	IQR	Median	SD	IQR	Median	SD	IQR	Median	SD	IQR	Median	SD	IQR
Hoop 1	70.00	66.84 ± 74.06	104.00	371.50	404.75 ± 383.64	651.00	1.0	0.91 ± 0.88	1.75	3.0	2.70 ± 2.23	4.00	70.0	108.32 ± 139.97	168.50	1062.0	833.14 ± 670.67	1391.25	75.0	116.75 ± 142.36	183.50	1087.0	845.86 ± 679.32	1393.75
Hoop 2	70.0	107.89 ± 118.34	188.0	190.0	303.71 ± 278.45	391.0	1.0	0.77 ± 0.86	2.00	2.0	1.26 ± 1.79	3.00	70.0	527.67 ± 519.59	283.00	551.0	104.51 ± 137.99	932.00	110.0	57.54 ± 69.04	283.00	551.0	68.91 ± 64.93	972.00
Hoop 3	62.0	71.26 ± 75.52	124.0	190.0	251.05 ± 236.77	481.0	1.0	0.86 ± 0.96	1.00	2.0	2.12 ± 1.69	3.00	62.0	95.74 ± 130.53	130.00	321.0	585.67 ± 623.96	1082.00	78.0	110.49 ± 153.47	170.00	351.0	105.51 ± 135.47	1142.00
Ball 4	0.0	39.67 ± 68.96	67.0	73.0	39.67 ± 68.96	376.0	0.0	0.56 ± 0.58	1.00	1.0	1.58 ± 1.91	3.00	0.0	339.38 ± 463.21	69.75	73.0	339.38 ± 463.21	665.75	0.0	350.92 ± 477.73	80.00	73.0	86.10 ± 173.57	685.75

Median = 50th percentile; SD, mean ± standard deviation; IQR, interquartile range.

**Table 6:** KW–H and Bonferroni results for AOI eye-tracking metrics across throw–catch phases.

Comparison			Hoop 1 vs Hoop 2	Hoop 1 vs Hoop 3	Hoop 1 vs Ball 4	Hoop 2 vs Hoop 3	Hoop 2 vs Ball 4	Hoop 3 vs Ball 4	*H*	*p*	*η^2^*
Total fixation duration	Throw	*z*	−1.372	−0.089	2.294	1.321	3.268	2.777	14.285	0.003	0.053
		*p*	0.164	0.929	0.013	0.179	0.001	0.003			
		*d*ᶻ	−0.35	−0.02	+0.59	+0.34	+0.84	+0.72			
		*r*	0.093	0.006	0.156	0.09	0.222	0.189			
	Catch	*z*	0.854	1.658	2.85	0.589	2.142	2.034	10.475	0.015	0.035
		*p*	0.392	0.095	0.003	0.555	0.028	0.038			
		*d*ᶻ	+0.22	+0.43	+0.74	+0.15	+0.55	+0.53			
		*r*	0.058	0.113	0.193	0.04	0.145	0.138			
Number of visits	Throw	*z*	−1.209	0.305	2.041	1.675	3.064	2.258	12.693	0.005	0.046
		*p*	0.204	0.745	0.026	0.072	0.001	0.014			
		*d*ᶻ	−0.31	+0.08	+0.53	+0.43	+0.79	+0.58			
		*r*	0.082	0.021	0.139	0.114	0.208	0.153			
	Catch	*z*	0.602	1.245	2.471	0.406	2.017	2.063	8.545	0.036	0.026
		*p*	0.544	0.206	0.011	0.681	0.038	0.034			
		*d*ᶻ	+0.16	+0.32	+0.64	+0.10	+0.52	+0.53			
		*r*	0.041	0.085	0.168	0.028	0.137	0.14			
Total duration of visit	Throw	*z*	−1.0363	0.408	2.17	1.343	2.96	2.482	11.912	0.008	0.042
		*p*	0.294	0.676	0.019	0.171	0.002	0.009			
		*d*ᶻ	−0.27	+0.11	+0.56	+0.35	+0.76	+0.64			
		*r*	0.07	0.028	0.147	0.091	0.201	0.169			
	Catch	*z*	1.673	1.693	3.427	0.103	2.393	2.553	14.747	0.002	0.055
		*p*	0.093	0.088	0.000	0.92	0.014	0.009			
		*d*ᶻ	+0.43	+0.44	+0.88	+0.03	+0.62	+0.66			
		*r*	0.114	0.115	0.233	0.007	0.162	0.173			
Total duration of glances	Throw	*z*	−1.031	0.359	2.107	1.463	2.9	2.386	11.479	0.009	0.040
		*p*	0.296	0.714	0.023	0.136	0.002	0.012			
		*d*ᶻ	−0.27	+0.09	+0.54	+0.38	+0.75	+0.62			
		*r*	0.07	0.024	0.143	0.099	0.197	0.162			
	Catch	*z*	1.633	1.624	3.368	0.0229	2.376	2.641	14.631	0.002	0.055
		*p*	0.101	0.102	0.001	0.984	0.015	0.007			
		*d*ᶻ	+0.42	+0.42	+0.87	+0.01	+0.61	+0.68			
		*r*	0.111	0.11	0.229	0.001	0.161				

*z*, standardized statistic from the Wilcoxon signed-rank test; *p*, Bonferroni-adjusted two-sided *p*-value; *d*ᶻ, within-subject effect size; *r*, rank-based, correlation-type effect size; *H*, Kruskal–Wallis omnibus test statistic.

#### 3.3.1 Total fixation duration in areas of interest

The results of the KW-H significance test indicated that both movement difficulty and apparatus type were significantly associated with the total fixation duration in visual search. The test for the throwing AOI yielded (*p* = 0.003, *η*^2^ = 0.053), and for the catching AOI, (*p* = 0.015, *η*^2^ = 0.035). Bonferroni *post hoc* pairwise comparisons further revealed that, during both the throwing and catching phases, the total fixation duration for hoop routines was significantly higher than that for Ball 4 (*p* < 0.05).

#### 3.3.2 Number of visits in areas of interest

The KW-H significance test showed that both movement difficulty and apparatus type significantly influenced the number of visits during visual search. The throwing AOI test produced (*p* = 0.005, *η*^2^ = 0.046), while the catching AOI test produced (*p* = 0.036, *η*^2^ = 0.026). Bonferroni *post hoc* analysis confirmed that, in both throwing and catching phases, the number of visits during hoop routines was significantly higher than during Ball 4 routines (*p* < 0.05).

#### 3.3.3 Total duration of visit in areas of interest

According to the KW-H significance test, both movement difficulty and apparatus type were significantly related to the total duration of visit. For the throwing AOI (*p* = 0.008, *η*^2^ = 0.042), and for the catching AOI (*p* = 0.002, *η*^2^ = 0.055). Bonferroni post hoc comparisons indicated the following: Throwing Phase: The total duration of visit in Hoop 1 and Hoop 3 was significantly lower than in Ball 4 (*p* < 0.05), while Hoop 2 was significantly higher than Ball 4 (*p* < 0.05). Catching Phase: The total duration of visit in Hoop 1 and Hoop 3 was significantly higher than Ball 4 (*p* < 0.001 and *p* < 0.05, respectively), whereas Hoop 2 was significantly lower than Ball 4 (*p* < 0.05).

#### 3.3.4 Total duration of glances in areas of interest

The KW-H significance test indicated that both movement difficulty and apparatus type were significantly associated with the total duration of glances in rhythmic gymnasts’ visual search behavior. For the throwing AOI, the test yielded (*p* = 0.009, *η*^2^ = 0.040), and for the catching AOI, (*p* = 0.002, *η*^2^ = 0.055). Bonferroni *post hoc* pairwise comparisons showed the following: Throwing Phase: The total duration of glances for hoop routines was significantly lower than that for the ball routine (*p* < 0.05). Catching Phase: Hoop 1 and Hoop 3 had significantly higher total duration of glances than Ball 4 (*p* < 0.001 and *p* < 0.05, respectively), while Hoop 2 had a significantly lower total duration of glances than Ball 4 (*p* < 0.05).

## 4 Discussion

The results indicate that, as task difficulty increases, elite athletes exhibit an overall upward trend in Total duration of whole fixations, Number of whole fixations, and Number of saccades. Comparing apparatus at the same difficulty level showed that the ball condition yielded significantly shorter total fixation duration and fewer fixation points than the hoop. At the areas of interest (AOI) level, effect sizes were predominantly small—medium (*η*^2^ ≈ 0.026–0.055), whereas overall indices were medium—large (*η*^2^ ≈ 0.105–0.143) (Cohen, 2013), consistent with current findings. Two factors primarily account for the modest AOI-level effects: (1) because the sample consisted of expert athletes whose visuomotor control is near a “ceiling,” between-condition differences shrink, attenuating standardized effects (Fleddermann et al., 2023); (2) in choreographed, apparatus-manipulation tasks such as rhythmic gymnastics, regulation often manifests as subtle shifts in temporal windows. Strategy differences among high-level athletes typically present as “fine-tuning rather than sweeping change”: even when effect sizes are modest, the patterns are directionally consistent, replicate across metrics, and can be translated into actionable training details. By analyzing visual search strategies under different difficulty levels and apparatus conditions in expert rhythmic gymnasts, this study provides more targeted guidance for visual training in elite performers.

Although many competition throw-and-catch sequences begin after athletes have moved to a predetermined, stationary formation, their visual context still differs markedly from our single-athlete laboratory task: competition introduces dynamic interference from teammates’ displacements and posture changes, apparatus trajectories, music-driven timing and formation transitions, and audience/screen/background motion. These factors require athletes to divide attention between their own predicted catch AOI and team/space-referenced AOIs, typically yielding increases in Number of saccades and Number of Visits and a relative shortening of Total duration of whole fixations on the key AOI. Accordingly, we adopted a stationary + single-athlete + single-apparatus paradigm to control interference and isolate mechanisms; the ecological validity of our findings therefore pertains primarily to isolated throw-and-catch segments rather than fully coordinated group routines.

### 4.1 Analysis of overall eye-tracking indicators during apparatus throw-and-catch in rhythmic gymnastics

This study adopted a throw-and-catch task replicating competitive routines. Through comparative analysis, it was found that as the movement difficulty increased, expert athletes exhibited a general upward trend in total duration of whole fixations, number of whole fixations, and number of saccades. Elite athletes typically adopt more efficient visual search strategies during movement execution. To ensure the precise execution of actions, athletes require longer total fixation durations to process visual information received (Tanaka et al., 2006). The movement “Hoop 3,” rated at a 0.4 high difficulty level, demonstrated significantly higher total duration of whole fixations compared to “Hoop 1” and “Hoop 2.” Complex movement sequences demand more precise visual tracking abilities (Young and Hulleman, 2013) to accurately judge apparatus positioning and release timing, ensuring successful execution. As “Hoop 3” involves ground-level rotational postures, athletes must rapidly locate and adjust to catch the target apparatus accurately after completing complex movements. This demands adjustments to apparatus position, speed, and optimal catching timing, thereby increasing the total duration of whole fixations. This phenomenon can be explained by cognitive load theory, which posits that higher cognitive load during complex catching tasks requires greater allocation of cognitive resources (Plass et al., 2010). Although “Hoop 3” and “Ball 4” share the same difficulty score, athletes show differences in their visual search processes. According to the common coding theory (Prinz, 1984), athletes activate motor patterns associated with the catching event during the ball’s flight phase. Therefore, expert athletes can anticipate the ball’s landing point in advance, thereby reducing cognitive processing load.

The shorter total fixation durations observed under higher-difficulty conditions can also be interpreted through predictive coding theory. This theory posits that the brain continuously generates predictions about upcoming sensory input and updates them by minimizing prediction error. During high-difficulty apparatus exchanges, athletes may rely more on feedforward predictions of apparatus trajectories and teammates’ actions, thereby reducing the need for prolonged fixations to acquire additional sensory information. Such anticipatory gaze strategies allow attentional resources to be allocated more efficiently to the preparation of subsequent motor actions. This mechanism is particularly relevant to the bonus elements specified in the International Gymnastics Federation (FIG) “Rhythmic Gymnastics Code of Points”—for example, throws combined with body rotations, out-of-sight, and non-hand catches—which require precise temporal coordination and efficient visual search to ensure successful execution. Accordingly, the shorter total fixation durations observed under high-difficulty conditions may reflect a shift toward greater automaticity in visual behavior—a shift that facilitates the completion of high-value bonus elements and, ultimately, contributes to higher competition scores.

The shorter fixations and faster transitions between areas of interest from the throw to the catch observed under higher-difficulty conditions can be regarded as manifestations of athletes’ perceptual–cognitive abilities. From the perspective of knowledge structures and long-term working memory, expert athletes can rapidly access task representations for apparatus trajectories, teammates’ movements, and musical timing, thereby reducing unnecessary movements and improving encoding efficiency; consistent with quiet eye research, stabilizing gaze on the anticipated catch location before the catch helps calibrate movement timing under uncertainty. These differences accord with contemporary attention theory: top-down attentional control and priority-based selection focus attention on task-relevant information while suppressing interference from rotational occlusion and teammates’ movements; rhythm-synchronized temporal attention narrows the effective sampling window prior to the catch, thereby enhancing precision of execution.

Previous studies have shown that under repeated conditions, visual search efficiency is faster and more effective compared to randomized settings (Solman and Smilek, 2012). Through deliberate practice, rhythmic gymnasts can master the release angle and anticipate the landing point of the apparatus, enabling faster and more accurate perception of dynamic scenes. Easier search tasks lead to earlier decision-making (Field, 2009); thus, deliberate practice is especially critical in training (Anders Ericsson, 2008). Long-term training can optimize athletes’ visual information processing capabilities (Faubert and Sidebottom, 2012), allowing for earlier anticipation of apparatus landing points and enhancing movement success rates. In this study, athletes were tested on movements of varying difficulty using the same apparatus. As task difficulty increased, cognitive load increased correspondingly, leading to a rise in the number of whole fixations and number of saccades among expert athletes.

### 4.2 Analysis of AOI eye-tracking indicators in apparatus throw-and-catch among rhythmic gymnasts

This study found that expert rhythmic gymnasts employed a highly efficient and experience-integrated visual strategy when performing throw-and-catch tasks of varying difficulty levels and apparatus types. When facing increased task difficulty or apparatus changes, athletes tended to shorten total fixation duration, reduce the number of fixations, and decrease total duration of glances, enabling quicker judgment of the apparatus trajectory and landing point. At the same time, high-difficulty movements prompted athletes to increase the overall number of saccades. These changes in visual search strategies reflect the athletes’ rapid adaptability to task load demands, aligning with the predictions of both the “Common Coding Theory” and “Cognitive Load Theory.”

The total fixation duration, number of fixations, total duration of visits, and total duration of glances for the hoop were significantly higher than those for the ball. As task difficulty increased, all four eye-tracking indicators associated with the hoop—total fixation duration, number of fixations, total duration of visits, and total duration of glances—exhibited a decreasing trend. Although the total duration of throw-and-catch remained relatively consistent across difficulty levels, the execution of higher-difficulty movements typically required more efficient visual search strategies. Although the total duration of throw-and-catch remained relatively consistent across difficulty levels, the execution of higher-difficulty movements typically required more efficient visual search strategies. Within the complete throw-and-catch sequence, gymnasts devoted more visual attention to the catching phase. This is likely because the success of the catch is directly tied to the overall success rate of the routine. This finding aligns with the Common Coding Theory (Prinz, 1990), which posits that athletes enhance movement accuracy by matching incoming sensory input during anticipation and catching with pre-existing action representations or memory codes. The observed decline in fixation-related indicators with increasing task difficulty may be attributed to the need for gymnasts to immediately transition into dynamic rotational movements after completing the throw in order to earn bonus points. Consequently, during the catching phase, the total fixation duration, number of fixations, total duration of visits, and total duration of glances all decreased. In the visual search process, it is possible to gather relevant target information at a fixation point prior to reaching the actual target. The final fixation before action serves to increase confidence rather than enhance success rate, indicating that this last fixation plays a unique role in boosting psychological certainty (Kotowicz et al., 2010). Therefore, rhythmic gymnasts must adopt faster and more efficient visual search strategies during the catching phase to ensure execution accuracy.

Although Hoop 3 and Ball 4 have the same difficulty score, the visual search strategies employed during the throw-and-catch actions differed. During the catching phase, expert athletes extracted the ball’s flight information and completed the catch by shortening total fixation durations, reducing the number of fixations, and minimizing saccadic paths. The shorter total fixation duration and fewer fixations indicate that athletes relied on a rapid visual search strategy after completing the throwing action, which aligns with the findings of Gabriel J. Diaz: occluded trajectory information may not be essential for subsequent visual search, and participants are likely to use predictive information about the ball’s trajectory to guide their gaze behavior. These results further support the role of memory in visual search processes (Diaz et al., 2013). The athletes’ visual search behavior was more focused and efficient, reducing unnecessary consumption of visual resources (Meghanathan et al., 2015).

Elite rhythmic gymnasts adopt a goal-directed visual search pattern and cognitive processing strategy (Vaeyens et al., 2007). The findings of this study highlight the benefits of integrating motor training with sports vision training. Based on the findings of this paper, “task difficulty and apparatus type systematically influence fixation patterns”, increased difficulty is accompanied by longer total duration of whole fixations and more focused AOI access; hoops demonstrate greater stability than balls across several fixation metrics. Routine training may employ adjustable parameters such as height/airtime, apparatus-body rotation, landing point deviation, partial occlusion, and catch constraints (single-handed/backward/shifting stance) to progressively construct scenarios: first establish stable “anticipatory look-back – steady fixation on projected landing AOI” using hoops, then transfer to balls to increase positioning challenges. Alter only one variable per session. By implementing sport-specific visual training tasks, athletes are more likely to adopt more efficient visual search patterns when processing and interpreting spatial information (Krzepota et al., 2015). Consequently, such training contributes to enhancing their spatial localization ability as well as their overall athletic performance.

## 5 Limitations and future research directions

This study has several limitations that should be addressed in future research: (1) The present study focused solely on expert athletes and did not compare visual search strategies between novices and experts. Future research should include athletes of varying skill levels to explore their distinct characteristics and developmental patterns, thereby informing tiered instruction and training design. (2) The tasks in this study involved only vertical-axis rotational difficulties and did not account for other bonus-scoring elements (e.g., floor positions, non-hand catches, visual occlusion). Future studies could incorporate a broader range of movements and apparatus conditions to provide a more comprehensive analysis of visual search strategy variations. (3) The current eye-tracking system may yield errors during rapid, large-amplitude movements, and is limited in sampling rate and field of view. Future research could employ higher-performance eye-tracking systems and 3D motion capture technology to improve data accuracy and ecological validity. (4) Limitations of behavioral indicators. The present study did not concurrently record athletes’ behavioral outcomes such as movement success rate and apparatus displacement, limiting a direct test of the relationship between visual search and behavioral performance. Future work using the same paradigm should concurrently record catch success/error rates and associated deductions, and relate these to key visual metrics.

## 6 Conclusion

This study examined expert female rhythmic gymnasts performing single-athlete throw-and-catch under graded difficulty and different apparatus using mobile eye-tracking. As task difficulty increased, athletes exhibited reduced total fixation duration, fewer fixation points, shorter Total duration of visit, and decreased total saccade duration during the apparatus-catching phase. Expert athletes tended to adopt anticipatory strategies during ball-catching actions to improve the success rate of reception. These results have theoretical and practical implications for elucidating visual processing in sport expertise. We propose two drills: (1) hoop-focused occluded-trajectory prediction training–—artificially occluding segments of the apparatus flight path to train anticipatory gaze control and prediction accuracy; (2) ball-focused spatial localization and rapid hand-response training–—introducing unpredictable ball flight paths and catch locations to enhance spatial perception and shorten visuomotor reaction time. Embedding these progressions into daily practice can help transfer laboratory-identified gaze strategies to competition settings.

## Data Availability

The datasets presented in this study can be found in online repositories. The names of the repository/repositories and accession number(s) can be found at: https://osf.io/vqs9f/files/osfstorage.
